# 577 Impact of the Bismuth Tribromophenate Stick-down Method on Pediatric Burns

**DOI:** 10.1093/jbcr/irad045.172

**Published:** 2023-05-15

**Authors:** Nikita Joshi, Jennifer Grauberger, Alex Joo, Janice Lalikos

**Affiliations:** UMass Chan Medical School, Worcester, Massachusetts; UMass Chan Medical School, Worcester, Massachusetts; UMass Chan Medical School, Worcester, Massachusetts; UMass Chan Medical School, Worcester, Massachusetts; UMass Chan Medical School, Worcester, Massachusetts; UMass Chan Medical School, Worcester, Massachusetts; UMass Chan Medical School, Worcester, Massachusetts; UMass Chan Medical School, Worcester, Massachusetts; UMass Chan Medical School, Worcester, Massachusetts; UMass Chan Medical School, Worcester, Massachusetts; UMass Chan Medical School, Worcester, Massachusetts; UMass Chan Medical School, Worcester, Massachusetts; UMass Chan Medical School, Worcester, Massachusetts; UMass Chan Medical School, Worcester, Massachusetts; UMass Chan Medical School, Worcester, Massachusetts; UMass Chan Medical School, Worcester, Massachusetts

## Abstract

**Introduction:**

Burn injuries are a leading cause of morbidity and mortality in the pediatric population. Patients require frequent dressing changes to minimize infection risk and promote wound healing via re-epithelialization. Bismuth Tribromophenate, an affordable petrolatum-based dressing, has been shown to have rapid re-epithelization of skin grafts and low infection rates in clean wound environments. A stick-down (SD) protocol has been developed to minimize the frequency of dressing changes. This SD protocol involves daily assessment and changing of the dressing until it begins to adhere to the underlying wound bed, where the dressing is then left in place as a protective layer as re-epithelialization occurs. The objective of this study is to characterize baseline features and compare outcomes associated with the SD dressing compared to non-stick-down (NSD) Bismuth Tribromophenate dressing.

**Methods:**

Patients under 18 years treated at our institution for partial thickness burn injuries were retrospectively identified using our medical records. Demographic data, injury characteristics, dressing type, length of hospitalization, and outpatient outcomes were collected from these records. Statistical analysis between dressing types was achieved using Fisher’s exact test and Mann-Whitney U test, with statistical significance defined as *p* < 0.05.

**Results:**

A total of 106 individuals receiving Bismuth Tribromophenate were identified. 36 patients received the Bismuth Tribromophenate SD dressing and 70 patients received NSD dressing. SD patients had a higher hospital admission rate compared to NSD patients (52.8% versus 33.3%, *p* = 0.088), though not statistically significant. 47.3% of admitted SD patients were admitted for burn severity compared to 25% in NSD patients. SD patients also had higher PICU admission rates compared to NSD patients (36.8% versus 4.8%, *p* = 0.017). SD patients also required initial debridement sedation more often (63.6% versus 36.7%, *p* = 0.017). SD and NSD patients had re-epithelialization rates of 66.7% and 65.6% (*p* = 1), respectively. Median time to re-epithelialization was 13 (10.8-17.2) days in SD patients compared to 12 (10-20) days in NSD patients (*p* = 1).

**Conclusions:**

SD-receiving patients were more likely to be admitted to the hospital and admitted for burn severity. In contrast, patients receiving NSD were less likely to be admitted, but more commonly admitted for pain. SD-receiving patients were also more likely to be admitted to the PICU and require sedation for debridement. Despite this difference in baseline characteristics, re-epithelialization rates and time to re-epithelialization between SD and NSD patients were not statistically distinct, with comparable rates and days to healing.

**Applicability of Research to Practice:**

Identifying cost-effective, efficacious, and convenient dressing protocols for the treatment of pediatric burns.

